# Chronic lithium administration in a mouse model for Krabbe disease

**DOI:** 10.1002/jmd2.12258

**Published:** 2021-11-12

**Authors:** Ambra Del Grosso, Gabriele Parlanti, Lucia Angella, Nadia Giordano, Ilaria Tonazzini, Elisa Ottalagana, Sara Carpi, Roberto Maria Pellegrino, Husam B. R. Alabed, Carla Emiliani, Matteo Caleo, Marco Cecchini

**Affiliations:** ^1^ NEST, Istituto Nanoscienze‐CNR and Scuola Normale Superiore, Piazza San Silvestro Pisa Italy; ^2^ Scuola Normale Superiore, Piazza dei Cavalieri Pisa Italy; ^3^ CNR Neuroscience Institute Pisa Italy; ^4^ Department of Chemistry, Biology, and Biotechnologies University of Perugia Perugia Italy; ^5^ Department of Biomedical Sciences University of Padua Padova Italy

**Keywords:** autophagy, globoid cell leukodystrophy, Krabbe, lithium, psychosine, Twitcher

## Abstract

Krabbe disease (KD; or globoid cell leukodystrophy) is an autosomal recessive lysosomal storage disorder caused by deficiency of the galactosylceramidase (GALC) enzyme. No cure is currently available for KD. Clinical applied treatments are supportive only. Recently, we demonstrated that two differently acting autophagy inducers (lithium and rapamycin) can improve some KD hallmarks in‐vitro, laying the foundation for their in‐vivo pre‐clinical testing. Here, we test lithium carbonate in‐vivo, in the spontaneous mouse model for KD, the Twitcher (TWI) mouse. The drug is administered ad libitum via drinking water (600 mg/L) starting from post natal day 20. We longitudinally monitor the mouse motor performance through the grip strength, the hanging wire and the rotarod tests, and a set of biochemical parameters related to the KD pathogenesis [i.e., GALC enzymatic activity, psychosine (PSY) accumulation and astrogliosis]. Additionally, we investigate the expression of some crucial markers related to the two pathways that could be altered by lithium: the autophagy and the β‐catenin‐dependent pathways. Results demonstrate that lithium has not a significant rescue effect on the TWI phenotype, although it can slightly and transiently improves muscle strength. We also show that lithium, with this administration protocol, is unable to stimulate autophagy in the TWI mice central nervous system, whereas results suggest that it can restore the β‐catenin activation status in the TWI sciatic nerve. Overall, these data provide intriguing inputs for further evaluations of lithium treatment in TWI mice.

**SYNOPSIS**

Here, we test lithium carbonate in‐vivo, in the spontaneous mouse model for Krabbe Disease, the Twitcher (TWI) mouse. Results demonstrate that lithium can slightly and transiently improves muscle strength, and restore the β‐catenin activation status in the TWI sciatic nerve.

## 1 INTRODUCTION

Globoid cell leukodystrophy, also called Krabbe disease (KD; OMIM #245200), is an autosomal recessive sphingolipidosis, belonging to the bigger class of lysosomal storage disorders (LSDs). It is included in the class of the rare diseases, having a worldwide incidence of about 1:100 000–1:250 000 live births.^1^ However, collectively, LSDs (about 90 clinically recognised metabolic disorders) can be classified as common, having an incidence of 1:5000 live births. For the most part, LSDs result from an enzyme deficiency within the lysosomes, which ultimately causes accumulation of undegraded substrates. KD is caused by homozygous or compound heterozygous mutations in the galactosylceramidase gene (GALC; OMIM #606890; chromosome 14q31), which causes decreased or abolished GALC enzymatic activity within cell lysosomes.^2^, ^3^, ^4^ Based on the Human Gene Mutation Database (HGMD), more than 130 KD‐associated mutations in the GALC gene have been identified, comprising deletions, frameshifts, and missense mutations.^5^ Depending on the specific mutations, different sub‐types of KD are recognised: infantile, late infantile, juvenile and adult.^6^

GALC is responsible for the degradation of the cytotoxic glycolipid called psychosine (PSY), whose accumulation is cytotoxic for the cell, and which has been found accumulated in both KD central and peripheral nervous system (CNS and PNS).^7^, ^8^, ^9^ This evidence paved the basis, in 1972, for the so‐called ‘PSY hypothesis’, which is the most accepted theory explaining KD pathogenesis.^10^ It has recently been confirmed by Li and colleagues.^11^ According to this theory, GALC deficiency leads to PSY intracellular accumulation, which primarily kills oligodendrocytes in the CNS^12^, ^13^ and Schwann cells in the PNS.^10^ Owing to the disruption of the neuronal‐glial homeostasis,^14^ these events cause demyelination and subsequent neurodegeneration. Further, the widespread cell death also leads to the activation of the inflammatory cascade, which recruits macrophages and activates microglia, amplifying the production of cytotoxic molecules.^15^ The inflammatory response to demyelination is then followed by astrocytic gliosis.^16^ In the areas of active demyelination, furthermore, multinucleated macrophages, called globoid cells, are often clustered around blood vessels.^17^, ^18^

The only available clinical treatment is haematopoietic stem cell transplantation (HSCT)^12^ for presymptomatic patients. In a few cases, this therapeutic approach could delay the onset and progression of the symptoms. Unfortunately, at the moment, no effective cure exists for KD. Available options are symptomatic and supportive only.^12^ A number of experimental treatments are currently under pre‐clinical studies, as gene therapy (GT),^19^ chaperone‐mediated therapy,^11^ or nanovector‐mediated enzyme replacement therapy (ERT).^4^, ^20^ Among them, GT has been the most investigated up to now, yielding good results in experimental models, such as mouse and dog.^19^, ^21^ In all studies, however, this approach did not allow completely curing the disease. Although GT was successful in increasing the GALC activity and reducing PSY accumulation, the lifespan of treated KD animals^22^, ^23^ could not reach the one of the non‐affected controls. This might be attributed to the fact that these therapies need time to engraft and create therapeutic effects, not allowing the effective prevention of the very early nervous system damage. Furthermore, molecular mechanisms other than PSY‐related cell death might play a role and be important to target during GT. In relation to this, it is worth noting, new aspects of the KD pathogenesis are emerging, as the presence of endothelial cell dysfunction,^24^ autophagy dysregulations,^25^, ^26^, ^27^, ^28^, ^29^ neuronal degeneration,^30^, ^31^, ^32^ and calcium signalling defects.^15^ Interestingly, it seems that these aspects cannot be effectively rescued by the only GALC supplying, suggesting the need for specific complementary therapies that could be associated with a GALC‐supplying therapy.

In line with this, we recently demonstrated the presence of autophagy dysfunctions in both in‐vitro and in‐vivo KD models.^27^, ^28^, ^29^ Autophagy is a dynamic degradation pathway by which cytosolic material, including damaged organelles, proteins, carbohydrates, and lipids, is delivered to the lysosome for degradation. Autophagy dysfunctions in KD have been confirmed also by other groups.^26^, ^33^ Generally, a dysregulation at the level of the main autophagy markers, as the microtubule‐associated protein 1 light‐chain 3 (LC3‐II),^26^, ^27^, ^28^ the ubiquitin‐binding protein p62 (p62),^27^, ^33^ Beclin‐1^27^ and the autophagy related 5 (ATG5)^29^), has been found. Data suggest the need of an up‐regulation of the autophagy flux in KD models, which is actuated from the cells as a physiological response to an increased rate of material to be degraded. Additionally, the presence of larger p62‐tagged aggregates in the brain of a KD mouse model suggests the concomitant inability of the KD cells to dispose‐off all the ‘waste materials’, indicating a possible engulfment of the KD autophagy flux.^27^, ^33^

Recently, we demonstrated^28^ that lithium can recover cell viability in a KD oligodendrocytes cell model via autophagy activation.^28^ Lithium, a drug clinically used to treat bipolar disorders, is an autophagy modulator acting via a mammalian target of rapamycin (mTOR)‐independent pathway. Moreover, lithium is able to induce the clearance of accumulated molecules in a number of neurodegenerative diseases (e.g., the mutant Huntingtin, tau protein, and alpha‐synuclein).^34^ It is noteworthy that alpha‐synuclein aggregates have just been recently found accumulated in KD human and mouse brains.^31^, ^32^, ^35^, ^36^

Therefore, here, we test the autophagy inducer lithium in a KD in‐vivo model, intending to rescue the phenotype by promoting the clearance of the undegraded autophagy substrates. We treated the Twitcher (TWI) mouse, the spontaneous and most widely used mouse model for KD,^37^ with lithium carbonate in the drinking water ad libitum, from post natal day (PND) 20. We longitudinally monitored the animal motor abilities, which normally result highly deteriorated in TWI mice with the progression of the disease.^19^ Specifically, we performed the grip strength test to measure the neuromuscular function, the hanging wire test to evaluate the muscular strength, and the rotarod test to evaluate the general motor coordination.^38^ Additionally, we measured a series of biochemical parameters, such as GALC enzymatic activity, PSY accumulation, and the astrogliosis status in the CNS and PNS. Finally, we investigated the two pathways potentially activated by lithium: the autophagy pathway, by looking at LC3‐II and p62 expression levels, and the β‐catenin (β‐Cat)‐dependent pathway, by looking at the expression of total and active β‐Cat levels.^29^

## 2 MATERIAL AND METHODS

### 2.1 Animal procedures

Twitcher heterozygous (HET) mice (TWI+/− C57BL6 mice; the Jackson Laboratory, Bar Harbor, ME), kindly provided by Dr. Biffi (San Raffaele Telethon Institute for Gene Therapy, Milan, Italy), were used as breeder pairs to generate homozygous TWI mice (TWI−/−, elsewhere in the paper abbreviated as TWI for simplicity) at the Center for Experimental Biomedicine of CNR, in Pisa. Animals were maintained under standard housing conditions and used according to the protocols and ethical guidelines approved by the Ministry of Health (permit no. 535/2018‐PR; official starting date: 9 July 2018). As previously done by our group,^20^, ^27^, ^39^ mice genomic DNA was extracted from clipped tails PND 10–15 mice (EUROGOLD Tissue‐DNA Mini Kit, EUROCLONE) and the genetic status of each mouse was determined from the genome analysis of the TWI mutation, as reported from Sakai and co‐workers.^40^ TWI, wild type (WT), and HET animals were all used for the experiments, and the HET littermates were also retained for colony maintenance. Mice, starting from PND 20–22, received chronic lithium carbonate (Li_2_CO_3_) dissolved into the drinking water at a concentration of 600 mg/L.^41^, ^42^, ^43^ Mice were treated until they reached a ponderal weight loss ≥ 25% (i.e., PND 37 and PND 40 in TWI and TWI‐Li mice). At this point they were sacrificed (WT and HET control mice, instead, were sacrificed with the last sacrificed TWI mouse of the specific experiment). Mice were subjected to the behavioural tests every 5 days from the starting time of the treatment (PND 20–22 as Day 0). At the end of the experiment, mice were deeply anaesthetised with a urethane solution (0.8 mL/Hg; Sigma–Aldrich) and euthanized by decapitation.

Subsequently, the tissues were extracted from each mouse and processed. All procedures were made with maximal efforts to minimise mice suffering.

### 2.2 Motor behavioural experiments

**FIGURE 1:**
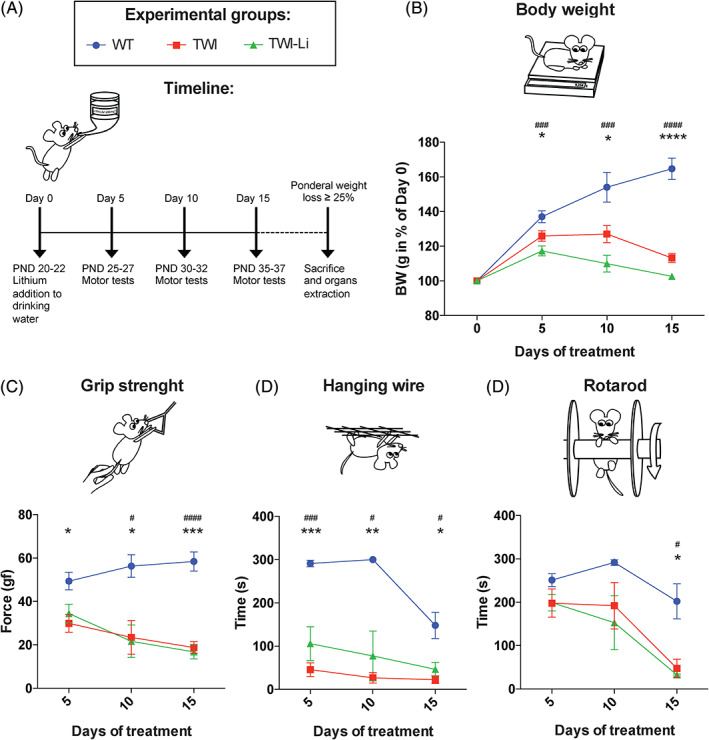
Administration of lithium carbonate by the drinking water in a mouse model of Globoid cell leukodystrophy. (A) Experimental design. Lithium carbonate (600 mg/L) has been administered via drinking water to the Twitcher (TWI) mice, starting at post natal day (PND) 20–22 (Day 0 of the experiments) and carry on until mice sacrifice (i.e., when ponderal weight loss ≥ 25%). As control, untreated Wilde type (WT) and TWI mice with similar age have been used for experiments. Motor tests (grip strength test, hanging wire test, and rotarod test) have been performed on treated (TWI‐Li) and untreated (WT, TWI) mice every 5 days after Day 0: Day 5, Day 10 and Day 15. (B–D) Lithium treatment effects on the mouse motor performances: measurements have been performed at Day 0, Day 5, Day 10 and Day 15 on WT (blue circles), TWI (red square), and TWI‐Li (green triangle) mice. (B) Body weight. Body weight (BW) has been measured (in grams) and reported in percent of BW at Day 0. * *p* < 0.05, ** *p* < 0.01 WT versus TWI; ### *p* < 0.001, #### *p* < 0.0001 WT versus TWI‐Li. (C) Grip strength test. Data are reported in the graph in gram‐force (gf). * *p* < 0.05, ** *p* < 0.01, *** *p* < 0.001 WT versus TWI; ## *p* < 0.001, ### *p* < 0.0001 WT versus TWI‐Li. (D) Hanging wire test. Data are reported in the graph in seconds (s). * *p* < 0.05, **** *p* < 0.0001 WT versus TWI; # *p* < 0.05, ## *p* < 0.01 WT versus TWI‐Li. (E) Rotarod test. Data are reported in the graph in seconds (s). * *p* < 0.05 WT versus TWI; # *p* < 0.05 WT versus TWI‐Li. (B–D) Data are reported as mean ± SEM and compared using One way ANOVA (Tukey post hoc test). *N* = between 3 and 10 mice for each experimental group

Motor behavioural experiments (wire hang, grip strength and rotarod test)^38^ were carried out every 5 days from the starting of the lithium treatment at Day 0 (so at Day 5, 10 and 15). Mice were divided in three experimental groups: WT untreated, TWI untreated and TWI mice treated with lithium (TWI‐Li; see Figure 1A for a graphical description of the experimental design). At each time point, the mouse body weight (BW) was also measured. For all motor behavioural experiments, groups of 3–10 mice have been tested, and data are reported as mean ±  standard error of the mean (SEM).

#### 2.2.1 Grip strength test

Mice were placed over a base plate in front of a grasping bar (trapezoid‐shaped) whose height was adjustable.^44^ The bar was fitted to a force transducer connected to a Peak Amplifier (Ugo Basile). When pulled by the tail, the animal instinctively grasped at the bar until the pulling force overcame their grip strength. After the animal lost its grip on the grasping bar, the peak amplifier automatically stored the peak pull‐force achieved by the forelimbs and showed it on a liquid crystal display. Three trials per day with an inter‐trial interval (ITI) of 15 min were performed for each animal and their average was calculated.

#### 2.2.2 Wire hang test

Mice were placed on a grid, which was then turned upside down and suspended 10–15 cm above the home cage.^45^ Two trials per day with an ITI of 15 min were performed for each animal and the mean latency to fall was calculated.

#### 2.2.3 Rotarod test

A rotarod apparatus (Ugo Basile) was used to assess the motor coordination skills, as previously done.^44^ The rotarod apparatus consists of five 3 cm diameter cylinders, which are suitably machined to provide grip. Six 25 cm diameter dividers make for five lanes, each 5.7 cm wide, enabling five mice to be on the rotor simultaneously. Mice were placed on the rotating rod suspended horizontally at a height of 16 cm from the floor. Four trials per day with an ITI of 15 min and a cutoff of 5 min at 4 rpm speed were performed for each animal and the mean latency to fall was calculated.

### 2.3 Mice organs processing

Tissues (half brain and sciatic nerves) were extracted from each mouse and immediately put on 1.5 mL microcentrifuge tubes on ice. Tubes were previously put on ice with distinct volumes of RIPA buffer (R0278; Sigma–Aldrich), depending on the organ (500 μL for the half‐brain and 100 μL for the sciatic nerves). RIPA buffer was previously prepared with both a protease and phosphatase inhibitors cocktail [cOmplete (4693116001) and PhosSTOP (4906845001); Roche Diagnostics]. Organs were then homogenised on ice with pestles, centrifuged (15 000 g for 30 min, at 4°C) and tested for protein concentration by the micro‐bicinchoninic acid (BCA) Protein Assay Kit (Thermo Scientific Pierce). The BCA‐assay was done using 1 μL of the organ lysate diluted in 100 μL of sterile distilled water, in 96 well plates. Each sample was assayed in duplicate. Absorbance (562 nm) was read by using the GloMax Discover Microplate fluorescence reader (Promega). Organs lysates were stored at −80°C.

### 2.4 Psychosine quantification

PSY quantification was assessed on homogenate samples after a lipid extraction step, using Liquid Chromatography coupled with High‐Resolution Mass Spectrometry (LC/HRMS). The extraction was carried out according to the method described by Fuller et al.,^46^ with minor modifications. Briefly, each homogenate sample (12–100 μL) was diluted 4 times with a mixture 1:2 of Chloroform/Methanol containing the PSY‐d5 as Internal Standard at the concentration of 200 ng/mL. Samples were mixed for 20 min on a thermomixer (Euroclone T‐Shaker) at 1700 rpm and room temperature. Afterwards, proteins were sedimented by centrifugation at 13 000 rpm for 10 min at 4°C. The supernatant was transferred into glass autosampler vials and immediately subjected to LC/HRMS analysis. A lipidomic liquid chromatography method^47^ was used to achieve a wide separation of PSY from other lipid molecular species. Quantitative data were obtained by injecting 20 μL of extract samples on an Agilent 6530 Q‐TOF LC/MS (Agilent Technologies, Inc., Santa Clara, CA), by Data Dependent Acquisition. Acetate adducts of natural PSY and PSY‐d5 were monitored in negative ionisation mode with accuracy within ±2.5 ppm. The peak area was used for quantification. PSY concentration was expressed as pg/100 mg of wet tissue and reported in percentage in respect to WT values.

Deuterated Psychosine Quantitative Mass Spec Standard (Galactosyl(β) Sphingosine‐d5; PSY‐d5) was purchased from Avanti Polar Lipids. Water, acetonitrile, isopropanol and methanol LC/MS grade, chloroform, ammonium acetate and ammonium fluoride were purchased from Sigma–Aldrich.

### 2.5 GALC enzymatic activity assay

GALC enzymatic assay was carried out in the mouse brain lysates. We used the 6‐hexadecanoylamino‐4‐methylumbelliferyl‐b‐Dgalactopyranoside (HMU‐βGal) molecule, the fluorescent substrate currently used for the clinical diagnosis of KD.^48^ Briefly, 10 μL of each lysate was added to 20 μL of 50 mM HMU‐βGal substrate solution and incubated for 17 h at 37°C. The reaction was then stopped by adding a pH 10.7 buffer solution, and the fluorescent product (6‐hexadecanoylamino‐4‐methylumbelliferato, HMU) was read by using the GloMax Discover Microplate fluorescence reader (Promega). GALC activity (reported as nanomole of the fluorescent product obtained in 17 h of incubation per milligram protein extract: nmol/h/mg) was calculated by comparison with a standard curve previously obtained by measuring the fluorescence of different concentration of 4‐methylumbelliferatogalactosylceramidase (4‐MU) and reported in percentage of the WT activity.

### 2.6 Immunoblotting

Western blot was carried out on tissue lysates (brain and sciatic nerves), processed as previously described, and stored at −80°C. Samples were thawed and boiled in Laemmli buffer containing β‐mercaptoethanol (5% final concentration) for 5 min, centrifuged at room temperature and the supernatants were finally used for gel electrophoresis (SDS‐PAGE) or kept at −80°C until use. Five micrograms of tissue lysates were resolved by SDS‐PAGE using Gel Criterion XT‐Precast polyacrylamide gel 4–12% Bis‐Tris (3 450 123; Bio‐Rad) and subsequently transferred to nitrocellulose membranes as previously described.^27^, ^28^ Immunodetection was performed with the following primary antibodies: anti‐LC3B (1:1000, rabbit, ab48394 Abcam), anti‐p62 (1:800, mouse, ab56416 Abcam), anti‐myelin binding protein (MBP) (1:1000, mouse, Abcam #62631), anti‐Glial Fibrillary Acidic Protein (GFAP) (1:1000, mouse, Synaptic System #173211BT), anti‐Non‐phospho β‐Catenin (1:1000, rabbit, Cell Signaling #8814), anti‐β‐Catenin (1:1000, rabbit, Cell Signaling #8480), and anti‐ionised calcium‐binding adapter molecule 1 (Iba‐1) (1:500, mouse, Sigma–Aldrich #MAB92). On the following day, the membranes were incubated with the corresponding peroxidase‐linked secondary antibodies (goat anti‐rabbit or mouse IgG‐horseradish peroxidase [HRP] conjugate; 1:2500–1:3000, Bio‐Rad #1706516 and 1706515) and developed with Clarity enhanced chemiluminescent substrates (Bio‐Rad #1705061). The chemiluminescent signal was acquired with an ImageQUANT LAS400 scanner (GE Healthcare Life Science), and the density of immunoreactive bands was quantified by ImageJ (NHI). The membranes were then incubated with a stripping buffer containing β‐mercaptoethanol (62.5 mM Tris–HCl pH 6.8, 2% SDS, 100 mM 2‐Mercaptoethanol) and re‐incubated as above with anti‐actin (1:3000, mouse, Sigma–Aldrich; # A3853), anti‐tubulin (1:3000, mouse, Sigma–Aldrich #T6074) or anti‐glyceraldehyde 3‐phosphate dehydrogenase (GAPDH) (1:3000, mouse, Sigma–Aldrich #G8795) antibody, and then processed as above. The results were normalised to the actin, tubulin, or GAPDH content for each brain and sciatic nerve lysate and reported as a percentage in respect to the WT untreated condition.

### 2.7 Statistical analysis

Data are reported as mean ± SEM obtained from at least three independent samples/experiments. In figure legends, ‘*N*’ indicates the number of samples/experiments. Data were statistically analysed by using Prism 6.00 (GraphPad Software, San Diego, CA; RRID: SCR_002798). For parametric data, one‐way ANOVA (Tukey post‐hoc test) or Student's *t* test were used; the mean values obtained in each repeated experiment were assumed to be normally distributed about the true mean. If not differently specified, statistical significance refers to results for which *p* < 0.05 was obtained.

## 3 RESULTS

### 3.1 Oral lithium administration slightly improves neuromuscular function

We treated mice with lithium carbonate administered at the concentration of 600 mg/L in the drinking water ad libitum, from PND 20. Meanwhile, we tested untreated Wild type (WT) mice, untreated Twitcher (TWI) mice, and TWI mice treated with lithium (TWI‐Li) for their motor abilities every 5 days (at Day 5–10–15) from the start of the lithium treatment at Day 0 (see Figure 1A for a graphical description of the timeline of the experiments and Section 2 for further information). In order to evaluate the BW increase/decrease in respect to the first day of treatment, we measured the mouse BW before and during the motor tests. As expected, the BW of TWI mice did not show the physiological BW increase found for WT mice already at Day 5 (Day 0–Day 5 BW % increase: WT = 137 ± 3; TWI = 126 ± 3). Additionally, from Day 5 until sacrifice, TWI mice showed a progressive BW loss (Figure 1B). TWI‐Li mice did not show any BW rescue if compared to TWI mice. Rather, their average BW resulted slightly less than that of TWI mice, although this difference was not statistically significant (Figure 1B), indicating that mouse survival was not affected by lithium treatment.

To evaluate the neuromuscular function, we performed the grip strength test (Figure 1C). As expected, WT mice presented a significantly higher force in respect to TWI, and also to TWI‐Li mice. Indeed, we did not measure significant differences between TWI mice and TWI‐Li mice at any time point of the treatment. The maximal muscle strength force of the forelimbs was already significantly reduced in both TWI and TWI‐Li mice in respect to WT at Day 5 (*p* value < 0.05 TWI and TWI‐Li vs. WT, One way ANOVA) and it progressively worsened with time (*p* < 0.001 TWI and TWI‐Li vs. WT at Day 15, One way ANOVA; Figure 1C).

The hanging wire test is commonly employed to evaluate motor deficits in the rodent models of CNS disorders, and here employed to evaluate the general deficit in motor function with focus on muscle strength. Also in this case, WT mice performed significantly better with respect to both TWI and TWI‐Li mice (Figure 1D). However, this difference showed a slight variation with lithium treatment. Indeed, TWI‐Li mice exhibited intermediate mean values between WT and TWI at Day 5 and Day 10 of treatment, statistically significant at Day 5 if considering a confidence interval of 80% (*p* = 0.198 TWI‐Li vs. TWI, One way ANOVA).

The last test was the rotarod test, commonly used to evaluate the motor coordination in rodents. At Day 5 all mice groups performed similarly (Figure 1E). At Day 10, TWI and TWI‐Li mice started showing a decrease in their rotarod performance, which resulted significantly deteriorated in respect to WT only at Day 15 (*p* < 0.05 WT vs. TWI and TWI‐Li, One way ANOVA). The motor coordination response did not appreciably differ in TWI mice with or without lithium treatment.

Altogether, these results suggest that lithium treatment by drinking water administration has no major rescue effects on the TWI motor performance and BW, although a mildly improved hanging wire capability can be appreciated at Day 5 of treatment.

### 3.2 The impact of lithium on demyelination and gliosis

The effect of lithium on the TWI pathophysiology was measured exploring the myelination and gliosis in the CNS of WT, TWI and TWI‐Li mice.

**FIGURE 2:**
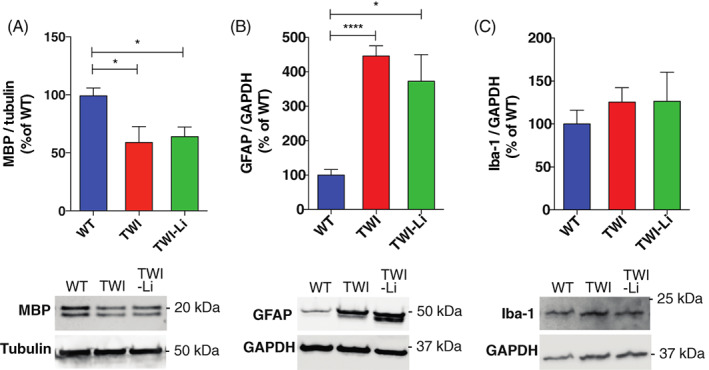
Lithium treatment in the Twitcher mice: the impact on brain demyelination and gliosis. Western blot analysis of different biomarkers in whole brain lysate of and untreated WT, TWI, and TWI lithium treated (TWI‐Li) mice. Under the graphs representative western blot bands are reported. (A) MBP. Western blot analysis of the total Myelin binding protein (MBP) content. * *p* < 0.05 TWI and TWI‐Li versus WT. *N* = between 4 and 9 mice for each experimental group. (B) GFAP. Western blot analysis of the total Glial Fibrillary Acidic Protein (GFAP). * *p* < 0.05 TWI‐Li versus WT; **** *p* < 0.0001 TWI versus WT. *N* = between 5 and 9 mice for each experimental group. (C) Iba‐1. Western blot analysis of the total Ionised calcium binding adaptor molecule 1 (Iba‐1) content. *N* = between 5 and 9 mice for each experimental group. All data are reported as mean ± SEM, normalised to the total Tubulin or Glyceraldehyde 3‐phosphate dehydrogenase (GAPDH) content, reported as percentage versus WT values, and compared using One way ANOVA (Tukey post hoc test)

Specifically, the demyelination status was quantified analysing the expression of MBP, a myelin marker whose decrease expression is generally associated with demyelinating diseases.^49^ We found a significant decrease (approximately 40%) in the expression of MBP in the brain of TWI mice in respect to what measured for WT mice (Figure 2A), and no effect of lithium treatment on the MBP expression in TWI‐Li (Figure 2A).

Another characteristic aspect of KD pathogenesis is gliosis, represented principally by astrocytosis and microgliosis.^50^ To investigate the effect of lithium on astrocytic gliosis, we quantified the expression of GFAP in the brain. GFAP is the main component of intermediate filaments of the cytoskeleton of astrocytes, and is usually found upregulated in case of demyelinating diseases.^51^ GFAP expression was increased (approximately 400%) in both TWI and TWI‐Li mice in respect to WT (Figure 2B). Microgliosis, which results in an increased number of activated microglia in the CNS,^52^ is especially involved in KD, since phagocytosis of myelin debris by microglia/macrophages results in the formation of globoid cells, one of the pathological hallmarks of the disease.^52^ As a microgliosis biomarker, we quantified Iba‐1, which is strongly and specifically expressed in microglia.^53^ As reported in Figure 2C, Iba‐1 expression showed an increasing trend in TWI and TWI‐Li vs. WT, without however reaching a statistical significance (*p* = 0.6 WT vs. TWI and TWI‐Li, One way ANOVA).

Overall, data suggest that the lithium treatment does not exert rescue effects on MBP, GFAP, and Iba‐1 expression in TWI mice, and therefore on myelination and gliosis.

### 3.3 GALC activity is not modified by lithium treatment

**FIGURE 3:**
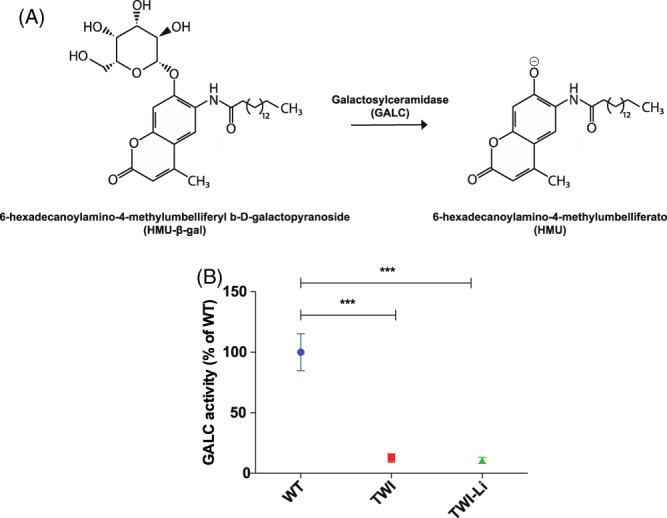
GALC activity following lithium administration. (A) GALC activity assay. The glycosidic bond of the substrate 6‐hexadecanoylamino‐4‐methylumbelliferyl b‐D‐galactopyranoside (HMU‐β‐gal) is cleaved by the GALC enzyme, resulting in the formation of the product 6‐hexadecanoylamino‐4‐methylumbelliferato (HMU), which is fluorescent at basic pH (10.7). (B) GALC enzymatic activity measured by HMU‐βGal assay in whole brain lysates of lithium treated (TWI‐Li) and untreated (WT, TWI) mice. Data are expressed in unit per microgram [(U/μg) = unit of enzyme per microgram of cell lysate; unit (U) = amount of enzyme that catalyses 1 nmol of substrate per hour] and reported in percentage of the WT activity. *** *p* < 0.001 TWI and TWI‐Li versus WT. Data are reported as mean ± SEM and compared using One way ANOVA (Tukey post hoc test). *N* = between 5 and 9 mice for each experimental group

In order to further characterise KD‐related biochemical parameters in the CNS, we also measured GALC enzymatic activity in the total brain lysates of WT, TWI and TWI‐Li mice. As already demonstrated, GALC enzymatic activity is approximately zero in the brain of the untreated TWI mice (Figure 3C), following the known fact that this naturally occurring mouse model contains a premature stop codon (W339X) in the GALC gene that abolishes the enzymatic activity.^40^ However, more recent findings have suggested that the suppression of the GALC expression in the TWI mouse is caused by a nonsense‐mediated mRNA decay (NMD).^54^ Lithium has been shown to influence the expression of hundreds of genes, although the mechanisms through which it regulates gene expression are still not completely understood.^55^ Moreover, data suggest that transcription factors and microRNAs, as well as epigenetic factors, may constitute key targets of lithium.^55^ For these reasons, we decided to check also the potential effects of lithium on GALC enzymatic activity in the TWI mice. As we observe in Figure 3C, GALC activity in the brain of TWI‐Li mice is equal to the activity of TWI mice (*p* < 0.001 TWI and TWI‐Li mice vs. WT, One way ANOVA), demonstrating that lithium does not interfere with the NMD process that leads to the complete abolition of the GALC activity.

### 3.4 The impact of lithium on the autophagy pathway

**FIGURE 4:**
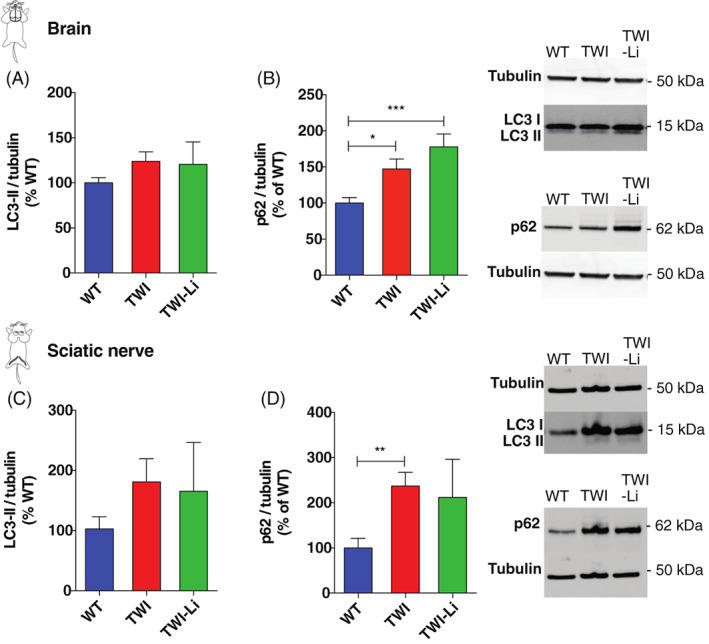
Lithium treatment in Twitcher mice: impact on the autophagy pathway. (A–D) Western blot analysis of the total autophagy‐related protein light chain 3 (LC3)‐II and sequestosome1/p62 (p62) content in whole brain and sciatic nerves lysates of lithium treated (TWI‐Li) and untreated (WT, TWI) mice. Representative western blot bands are reported on the right of the graphs. (A) LC3‐II/Brain. *N* = between 5 and 9 mice for each experimental group. (B) p62/Brain. * *p* < 0.05 TWI versus WT; *** *p* < 0.001 TWI‐Li versus WT. *N* = between 5 and 15 mice for each experimental group. (C) LC3‐II/Sciatic nerve. *N* = between 4 and 15 mice for each experimental group. (D) p62/Sciatic nerve. ** *p* < 0.01 P TWI versus WT. *N* = between 5 and 15 mice for each experimental group. All data are reported as mean ± SEM, normalised to the total Tubulin content, reported as percentage versus WT values, and compared using One way ANOVA (Tukey post hoc test)

To investigate the lithium effects on the autophagy pathway, we quantified the expression of two fundamental autophagy markers, LC3‐II and p62,^27^ in the brain and sciatic nerve of WT, TWI and TWI‐Li mice by western blot. Regarding the brain, we found an increased expression of LC3‐II in TWI in respect to WT mice (*p* < 0.5 TWI vs. WT, Student's *t*‐test; Figure 4A), confirming the hyperactivation of the autophagy flux previously found in in‐vitro and in‐vivo TWI models.^27^ Unexpectedly, we did not found any further increase in TWI‐Li mice (Figure 4A), differently from what we previously found in‐vitro.^28^ p62 was increased in the TWI brain (Figure 4B), confirming the previously published data,^27^, ^33^ and showed similar levels also in TWI‐Li mice (Figure 4B). The absence of p62 reduction in the brain confirms the data about LC3‐II, indicating that lithium treatment did not further stimulate autophagy in the TWI mice.

In the PNS we found similar results. LC3‐II, in fact, similarly increased in both TWI and TWI‐Li sciatic nerve, with respect to WT (Figure 4C,D), and the p62 level of TWI‐Li mice did not significantly decrease with respect to TWI level.

Overall, these data suggest that the implemented lithium treatment protocol could not boost autophagy in‐vivo in TWI mice, either in the CNS or PNS.

### 3.5 Psychosine accumulation

**FIGURE 5:**
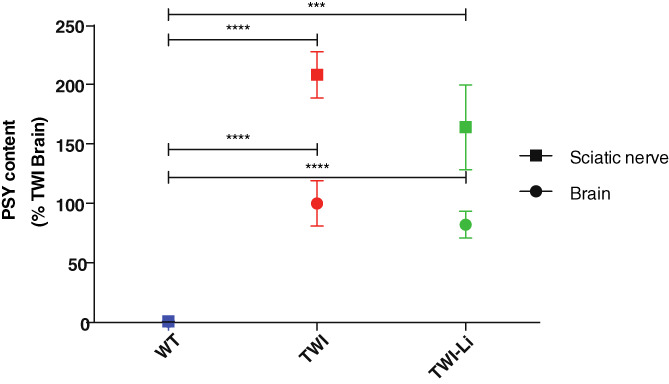
Psychosine (PSY) content accumulation in the central and peripheral nervous system of TWI mice after lithium treatment. HPLC/MS quantification of PSY content in lipid fraction extracted from whole brain and sciatic nerve lysates of lithium treated (TWI‐Li) and untreated (WT, TWI) mice. *** *p* < 0.001 P TWI‐Li versus WT (sciatic nerve); **** *p* < 0.0001 TWI versus WT (sciatic nerve and brain) and TWI‐Li versus WT (brain). *N* = between 4 and 11 mice (for sciatic nerve) and 5 and 9 mice (for brain), for each experimental group. Data are quantified as pg/100 mg of wet tissue, reported in percentage in respect to WT values as mean ± SEM, and compared using One way ANOVA (Tukey post hoc test)

To understand if the lithium treatment could affect PSY accumulation in TWI mice, we quantified the amount of PSY in the brain and sciatic nerve of WT, TWI and TWI‐Li mice. As reported in Figure 5A, PSY is greatly accumulated both in the TWI brain and sciatic nerve (*p* < 0.0001 TWI vs. WT, One way ANOVA), and it also resulted higher in the sciatic nerve with respect to the brain, as previously reported.^19^, ^22^ As expected, PSY concentration in the WT samples was undetectable (Figure 5A). Regarding both the brain and the sciatic nerve, TWI‐Li mice showed PSY concentration similar to that of TWI mice, demonstrating that our lithium treatment did not modify the PSY accumulation in the CNS and PNS.

### 3.6 The impact of lithium on β‐catenin phosphorylation

**FIGURE 6:**
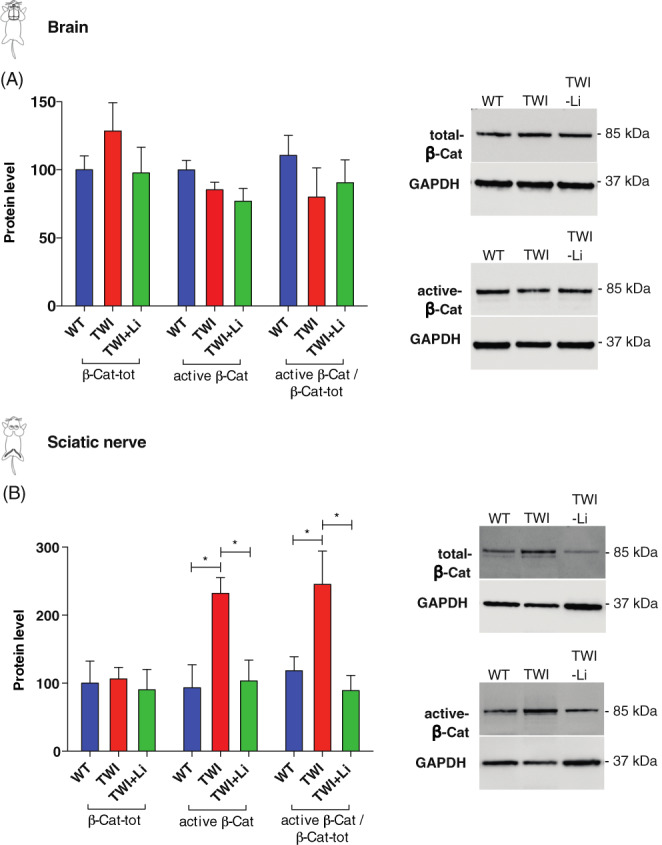
Effect of lithium on β‐catenin activation. (A,B) Western blot analysis of the total β‐catenin (phosphorylated and not phosphorylated forms) and active β‐catenin (not phosphorylated form) in whole brain and sciatic nerves lysates of lithium treated (TWI‐Li) and untreated (WT, TWI) mice. Active β‐catenin/total β‐catenin is also reported. On the right of the graphs are reported representative western blot bands. (A) Brain. (B) Sciatic nerve. * *p* < 0.05 TWI versus WT and versus TWI‐Li. *N* = between 5 and 9 mice for experimental group. All data are shown as mean ± SEM and compared using One way ANOVA (Tukey post hoc test)

To further investigate the effect of lithium treatment in TWI mice, we studied the β‐cat‐dependent signalling, which is known to be potentially affected by lithium.^19^ We measured the expression of total β‐Cat (phosphorylated β‐Cat and not phosphorylated β‐Cat), and active β‐Cat (not phosphorylated β‐Cat) in the brain and sciatic nerve lysates of WT, TWI and TWI‐Li mice, by western blot. As reported in Figure 6A, the amount of total β‐Cat and active β‐Cat and their ratio (active β‐Cat/total β‐Cat) is quite similar in the brain of WT, TWI and TWI‐Li mice. A mild decrease in the values of active β‐Cat/total β‐Cat is possibly appreciable. Differently, in the sciatic nerve, a clear effect of lithium treatment on β‐Cat activation was observed (Figure 6B). While the values for total β‐Cat remain stable between WT, TWI and TWI‐Li mice, the activation of β‐Cat was significantly increased in TWI mice with respect to WT (*p* < 0.05 TWI vs. WT, One way ANOVA). Interestingly, the value of active β‐Cat in the TWI‐Li mice returned to the WT level (*p* < 0.05 TWI‐Li vs. TWI, One way ANOVA). The same trend was reported for the ratio active β‐Cat/total β‐Cat.

These results suggest that, in the peripheral nervous system, the β‐cat‐mediated pathway is hyperactivated in the TWI mice, and that lithium treatment can restore its status at the WT level.

## 4 DISCUSSION

In this paper, we tested a lithium treatment protocol in‐vivo in the TWI mice. We investigated the effect of lithium on motor function and on biochemical parameters related to KD. We demonstrated that lithium has no major rescue effects on the TWI motor phenotype, although it shows a mild positive effect on the muscle strength at early time. We also proved that, with this administration protocol, lithium cannot modulate gliosis and demyelination, and stimulate autophagy in the TWI nervous system, whereas results suggest it can restore the β‐cat signalling in the TWI peripheral nervous system.

We chose to use lithium in the form of lithium Carbonate considering that it is already used in this form in the clinical practice for mental disorders (CARBOLITHIUM®). Specifically, we treated TWI mice with lithium 600 mg/L in the drinking water, a dose/route of administration that induced autophagy in other mouse models, and that has been demonstrated to be able to give a serum‐mice‐concentration similar to the human therapeutic one.^41^, ^42^, ^43^ We treated mice starting from PND 20, the time window when the first signs of KD manifestations occur in the TWI mice; the symptoms' onset is often the moment when the diagnosis is made and therapies can usually start in human patients.

We found no variations between the ability to perform the grip strength and the rotarod test of the untreated versus lithium‐treated TWI mice, indicating that lithium has no rescue effects on the neuromuscular force and on the general motor coordination behaviour. On the other hand, however, results of the hanging wire test suggest that lithium has a mild positive effect on muscle strength in the initial phase of the treatment. A slight improvement in the mean of hanging wire capability of TWI‐Li mice, in fact, is appreciable at Day 5 of the treatment (PND 25–‐27), whereas it completely vanished with the advancement of the disease (PND 35–37).

Subsequently, we investigated the expression of GFAP, MBP, and Iba‐1 in the brain lysates, in order to investigate the effect of lithium on the pathophysiological processes of myelination and gliosis. We did not found significant differences between the expressions of such markers between untreated and lithium‐treated TWI mice, indicating that lithium is not able to change the whole myelination and gliosis status in the TWI brain. These results are in accordance with the motor experiments ones, which showed no major changes in the performance of lithium‐treated mice. All the just above‐mentioned markers resulted significantly higher in TWI mice in respect of the healthy WT controls, confirming previous results.^29^, ^39^

In order to further characterise the KD‐related parameters in the brain of TWI‐Li mice, we also measured the GALC enzymatic activity. As already found, GALC enzymatic activity was approximately zero in the brain of the untreated TWI mice, in accordance with the fact that this mouse model contains a premature stop codon in the GALC gene that abolishes enzymatic activity.^40^ However, the choice to measure GALC activity in the brain of lithium‐treated TWI mice was driven by the fact that more recent findings suggest that the suppression of the GALC expression in the TWI mouse is caused by NMD,^54^ and that transcription factors and microRNAs, as well as epigenetic factors, may constitute key targets of lithium.^55^ Our data showed that GALC activity in the brain of lithium‐treated TWI mice is approximately equal to the activity of the untreated ones, demonstrating that lithium does not interfere with the NMD process.

Subsequently, we investigated the effects of lithium treatment at the molecular level on the autophagy pathway, by quantifying the expression of LC3‐II and p62. Both LC3‐II and p62 are in fact increased in the brain and sciatic nerve of TWI mice compared to WT, confirming results previously found by us and others.^27^, ^29^, ^33^ Surprisingly, we found no differences between the expressions of these markers upon lithium treatment, both in the brain and the sciatic nerve, suggesting that in‐vivo lithium is not able to boost autophagy, neither in the CNS nor in the PNS of the TWI mice.

As a likely consequence of and in accordance with this, we also found that PSY concentration in the brain and sciatic nerve of lithium‐treated mice was similar to the one of untreated TWI mice, meaning that lithium did not help with PSY clearance in the nervous system.

Finally, we investigated the other pathway that is activated by lithium, the β‐Cat‐mediated pathway. Evidence in the literature shows that the effect of lithium strictly depends on the administered dose. It is known that lithium can induce autophagy, by inhibiting phosphatidylinositol trisphosphate (PIP3) formation, at concentrations considered ‘low doses’ (for the specific model), whereas at ‘higher doses’ it inhibits autophagy, by increasing the concentration of the non‐phosphorylated β‐Cat (the active form), causing augmented β‐Cat‐mediated transcription.^56^ We found that the levels of β‐Cat activation remain approximately stable in the brain of WT and TWI mice (both treated and untreated). Importantly, instead, in the sciatic nerve the active β‐Cat level significantly increased in TWI untreated mice, while in the TWI mice treated with lithium returned to the WT basal levels. These results suggest that lithium treatment could restore the activation status of the β‐Cat‐mediated pathway in the TWI PNS. It is conceivable that a negative feedback is responsible to diminish the β‐Cat signalling in TWI‐Li mice during lithium treatment. β‐Cat‐mediated signalling, in fact, being already hyperactivated in TWI mice, could likely not be able to further increase in TWI‐Li, due to negative feedback mechanisms controlled by specific β‐Cat‐regulated genes.^57^, ^58^ Interestingly, hyperactivation of β‐Cat signalling, never reported up to known for KD, has already been found for Mucopolysaccharidosis type II, in which has been associated with the impaired atrioventricular canal differentiation.^59^ Furthermore, restoration of the β‐Cat‐mediated signalling is currently investigated for other neurodegenerative diseases, as Alzheimer's^60^ or Gaucher's disease,^60^ suggesting β‐Cat pathway as promising to be investigated in order to explore new KD therapeutic targets, in particular for the PNS.

Interestingly, while it is true that given that our data seems to exclude an effect of lithium on myelination and gliosis, they also suggest the possibility of a neuroprotective effect. Some type of diseases characterised by PNS defects, in fact, as neuropathy in diabetic patients or traumatic nerve injuries, show activation of the β‐cat pathway in the sciatic nerves, similarly to what found for TWI mice.^61^, ^62^ Additionally, some studies demonstrated the neuroprotective capability of β‐cat signalling inhibition.^62^, ^63^

Thus, looking at all the data together, we can hypothesize that the mild and transient improvement in the muscle strength of TWI‐Li mice cannot be correlated to the stimulation of the autophagy pathway, as we expected, but to the alteration of the β‐Cat‐mediated transcription.

Overall, the effect on the activation of the β‐Cat pathway, in association with the slightly improved muscle strength at short‐term, suggests lithium as a drug to be further investigated in the TWI mice. However, a refinement of the dose might be needed to optimise the approach. Some results, in fact, could be due to an underdosing of lithium, caused by concentrations and treatment time that could be not optimal for assessing its efficacy. Further studies will be needed to measure blood levels of lithium^42^ or monitor a lithium‐sensitive marker, such as an effect on circadian rhythm.^64^

The use of other administration routes for the lithium treatment could also be captivating. Administration via drinking water, in fact, is a convenient route for some reasons (easy modality of drug preparation and administration, no stressing for the animals), but it may lead to variability in the taken lithium dose between the mice. The TWI mice, which typically experience little differences in their pathological status, may differ in the water uptake (as previously reported for other mice strains^65^).

Starting the administration of lithium at neonatal period (PND 0–2) could also be interesting to test, although of minor relevance regarding a possible clinical translation. Indeed, the possibility that KD is diagnosed before symptoms onset in humans is very rare. Moreover, this approach should be carefully evaluated, because of the known side effect of lithium carbonate to cause teratogenicity during early development in animal models.^66^ The most serious side effect of lithium on nervous system development is the formation of neural tube defects (NTDs), including myelomeningocele, anencephaly, and encephalocele, but the occurrence rate and susceptibility to NTDs in mice exposed to lithium are significantly influenced by the uptaken dose of the drug and by the mouse strain.^67^

Currently, many therapeutic approaches for KD are currently under pre‐clinical studies, as GT (mainly based on adeno‐associated vector),^36^, ^68^ chaperone‐mediated therapies,^11^ or nanovector‐mediated ERT.^20^ Among them, GT demonstrated to be able to increase GALC levels in the TWI tissues up to the WT levels, to reduce the cytotoxic PSY‐accumulations, to improve the motor behaviour of TWI mice, and, very importantly, to extend the lifespan from ~45 days to ~500 to 700 days.^36^ Unfortunately, however, in all GT studies KD mice at a certain point died with uncertain complications.^36^, ^68^, ^69^, ^70^ Two very recently published papers suggest that GT could not be able to efficiently act on a long time due to the exhaustion of therapeutic AAV episomal DNA in specific regions,^23^ or to the onset of additional pathological events unrelated to KD, as hepatocellular carcinoma (HCC).^22^ Additionally, a very intriguing possibility is that there could be also additionally pathogenic mechanisms, not directly related to GALC deficiency, which could significantly contribute to the pathogenesis and the bad progression of KD. Thus, nowadays in the field it is overall accepted that there is a need for complementary therapies that can act synergistically with a main GALC‐deficiency correcting therapy, in order to restore the patient healthy state. Among mechanisms that have been recently demonstrated to be related to the KD pathogenesis, we previously investigated autophagy dysfunctions. We demonstrated the presence of autophagy dysregulations in both in‐vitro and in‐vivo KD models.^27^, ^28^ Moreover, we tested in‐vitro two differently‐acting drugs known to be autophagy inducers: lithium and rapamycin.^56^ Both drugs demonstrated to be able to promote the partial recovery of the TWI phenotype in immortalized^28^ and primary KD cell models,^27^ paving the basis for their in‐vivo pre‐clinical testing.

Thus, here, we provided for the first‐time data about in‐vivo lithium administration in the spontaneous mouse model for KD. Lithium is a well‐known drug already on the market, and its experimentation is especially encouraging, given that the clinical translation would be easily performed. Intriguingly, we obtained that lithium 600 mg/L administered ad libitum via the drinking water leads to minor rescue effects on the KD phenotype and does not induce autophagy activation in the TWI nervous system. Remarkably, our results suggest that lithium treatment can restore the hyperactivation of the β‐Cat‐mediated signalling in the PNS of TWI mice. Finally, our data suggest that the administration protocol and dosage could be further investigated.

## CONFLICT OF INTEREST

The authors declare that they have no competing interests.

## AUTHOR CONTRIBUTIONS

All authors had full access to all the data in the study and take responsibility for the integrity of the data and the accuracy of the data analysis. Study concept and design: Ambra Del Grosso, Lucia Angella and Marco Cecchini. Acquisition of data: Ambra Del Grosso, Gabriele Parlanti, Lucia Angella, Nadia Giordano, Elisa Ottalagana, Roberto Maria Pellegrino, Husam B. R. Alabed, Ilaria Tonazzini and Sara Carpi. Analysis and interpretation of data: All authors. Drafting of the manuscript: All authors. Statistical analysis: Ambra Del Grosso, Ilaria Tonazzini and Marco Cecchini. Obtained funding: Ambra Del Grosso and Marco Cecchini. Study supervision: Marco Cecchini, Matteo Caleo and Carla Emiliani.

## Supporting information


**Figure S1** Standard 4‐MU curve. Calibration curve which correlates increasing concentration of 4‐methylumbelliferatogalactosylceramidase (4‐MU) with the emitted fluorescence (FL).
**Figure S2.** Representative chromatograms with the PSY monitored transition for quantitative purpose.

## Data Availability

All data needed to evaluate the conclusions in the paper are present in the paper. Additional data related to this paper may be requested from the authors.
