# Supplementary material for: Chronic lithium administration in a mouse model for Krabbe disease
Source: JIMD Rep. 2021 Nov 12;63(1):50–65. doi: 10.1002/jmd2.12258 (PMC8743347; doi:10.1002/jmd2.12258)
Supplement: Supplementary file 1 — Figure S1 Standard 4‐MU curve. Calibration curve which correlates increasing concentration of 4‐methylumbelliferatogalactosylceramidase (4‐MU) with the emitted fluorescence (FL). Figure S2. Representative chromatograms with the PSY monitored transition for quantitative purpose. [file JMD2-63-50-s001.pdf]

Supplementary figures

Figure S1

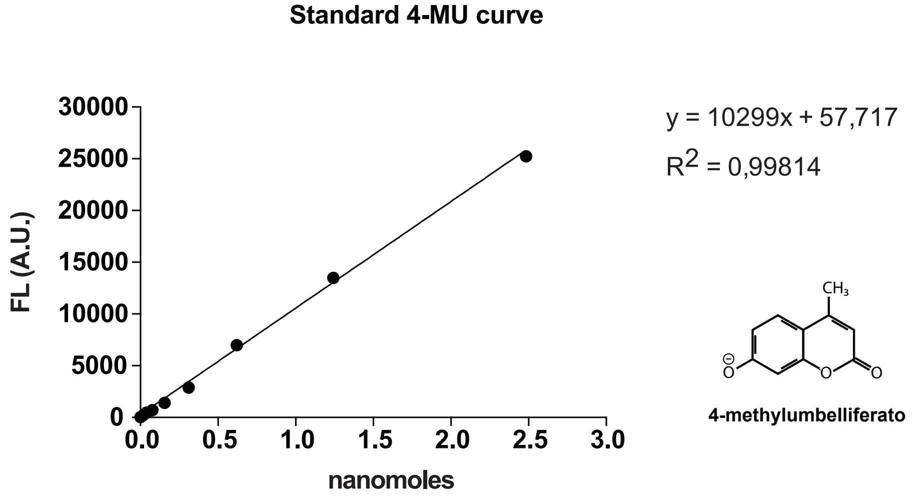

Figure S1. Standard 4-MU curve. Calibration curve which correlates increasing concentration of 4-methylumbelliferatogalactosylceramidase (4-MU) with the emitted fluorescence (FL).

Figure S2

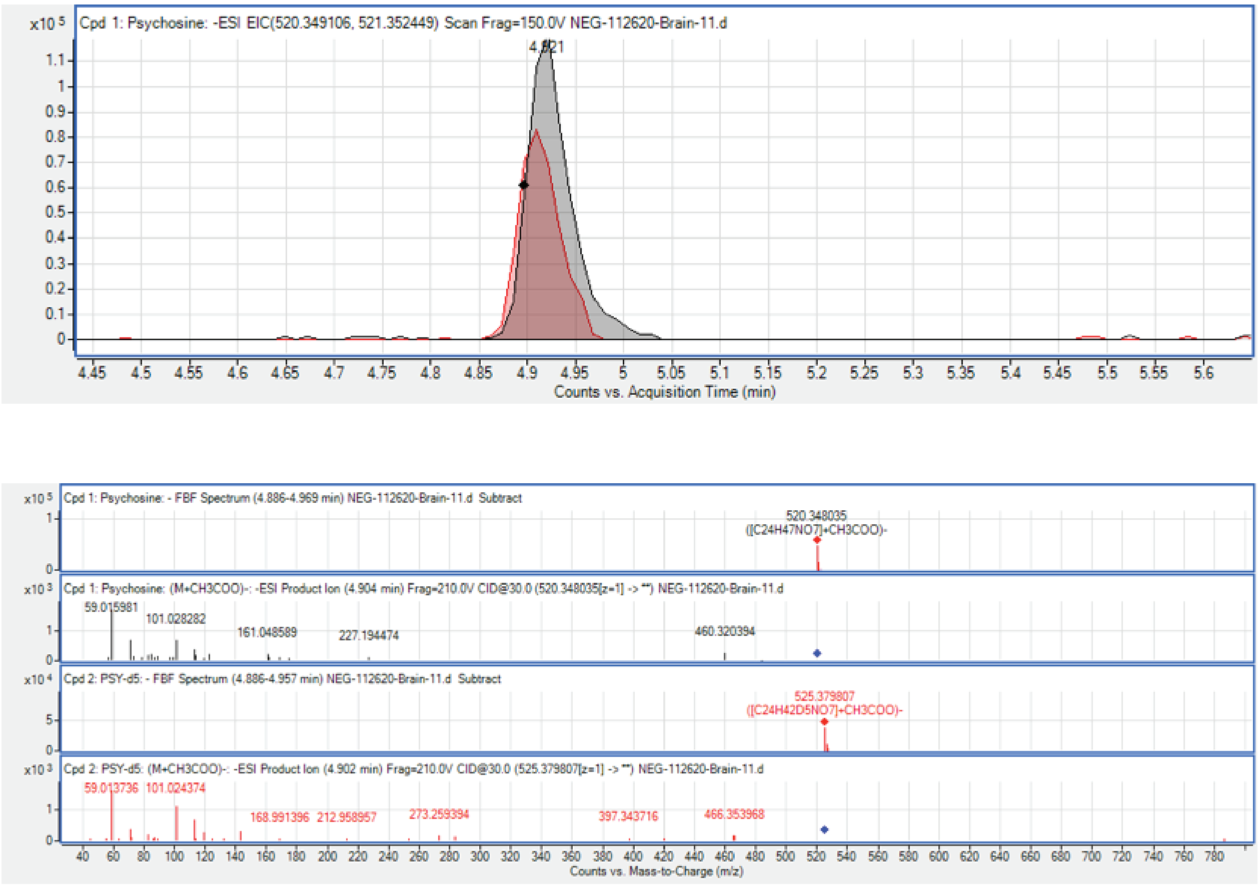

Figure S2. Representative chromatograms with the PSY monitored transition for quantitative purpose.
